# Association between referral source and duration of untreated psychosis in pathways to care among first episode psychosis patients in Northern Malawi

**DOI:** 10.1111/eip.12885

**Published:** 2019-10-27

**Authors:** Atipatsa C. Kaminga, Japhet Myaba, Wenjie Dai, Aizhong Liu, Harris K. Chilale, Paul F. Kubwalo, Precious Madula, Richard Banda, Xiongfeng Pan, Shi W. Wen

**Affiliations:** ^1^ Department of Epidemiology and Health Statistics, Xiangya School of Public Health Central South University Changsha China; ^2^ Department of Mathematics and Statistics Mzuzu University Luwinga Malawi; ^3^ Department of Clinical Medicine, Mental Health Research Section Saint John of God Community Services Mzuzu Malawi; ^4^ Department of Communication Studies Mzuzu University Mzuzu Malawi; ^5^ OMNI Research Group, Department of Obstetrics and Gynecology, Faculty of Medicine University of Ottawa Ottawa Ontario Canada; ^6^ Clinical Epidemiology Program Ottawa Hospital Research Institute Ottawa Ontario Canada; ^7^ School of Epidemiology, Public Health, and Preventive Medicine, Faculty of Medicine University of Ottawa Ottawa Ontario Canada

**Keywords:** duration of untreated psychosis, early intervention, first episode psychosis, pathways to care, schizophrenia

## Abstract

**Aims:**

To examine the association between referral source and duration of untreated psychosis (DUP) and explore determinants of referral source; when adjusting for pathways to care, positive and negative symptoms, diagnosis and socio‐demographic characteristics.

**Methods:**

A total of 140 subjects with first episode psychosis (FEP) were enrolled from a pilot early intervention service for psychosis in Northern Malawi between June 2009 and September 2012. Logistic regression analyses were used to quantify the associations between variables of interest.

**Results:**

Age ranged between 18 and 65 at assessment, with median, 33. Median DUP was 12.5 months. First contact did not independently determine DUP. Long DUP (>6 months) was associated with referral from community based volunteer (CBV) or traditional healer (TH), a unit increase in severity of negative symptoms and having schizophrenia, which was also associated with referral from CBV or TH. Additionally, being unemployed was associated with referral from CBV or TH. However, a unit increase in the number of times religious advice (RA) was sought, GP was contacted and severity of positive symptoms was associated with referral by GP.

**Conclusions:**

Mental health awareness is justified for this population and collaboration with THs in identifying and treating patients with psychosis may help reduce treatment delays. Access to mental health services ought to improve, particularly for the unemployed group. Future studies should consider adjusting for referral source when ascertaining first contact source as a predictor of DUP.

## 1 INTRODUCTION

Delays in receiving effective treatment are common among patients with first episode psychosis (FEP) (Norman, Malla, Verdi, Hassall, & Fazekas, 2004; O'Callaghan et al., 2010; Perkins, Nieri, & Penn, 2005) and these have been shown to have adverse repercussions on the prognosis of the disease (Marshall et al., 2005; Penttilä, Jääskeläinen, Hirvonen, Isohanni, & Miettunen, 2018), hence it has been deemed necessary to shorten duration of untreated psychosis (DUP), the time from manifestation of the first psychotic symptom to initiation of adequate treatment (Marshall et al., 2005). Among other factors, delays in referral to effective treatment have contributed to longer DUP (Birchwood et al., 2013; Brunet, Birchwood, Lester, & Thornhill, 2018). Thus, referral sources are very critical in the quest for early intervention services (EISs) to reduce DUP and pathways to psychiatric care, which describe a trail to receiving effective treatment, are indispensable in understanding referral delays in FEP. In other words, referral delay is a function of pathways to care.

A number of studies exploring pathways to care found that majority of the participants in FEP have first contact with GP (Bhui, Ullrich, & Coid, 2014; Temmingh & Oosthuizen, 2008). Besides, those having first contact with, or referrals from sources other than GP or specialists were more likely to have long DUP at receiving effective treatment (Bhui, Ullrich, & Coid, 2014; Cocchi et al., 2013). However, sources of contact or referral varied considerably across populations, particularly with respect to cultural differences on illness attributions among different ethnic groups (Anderson et al., 2015; Ferrari et al., 2015; Flora et al., 2017). Therefore, different ethnic groups may require different early interventions on pathways to care to reduce treatment delays. In this regard, an assessment of pathways to care for a specific ethnic group would be cost efficient when planning early interventions on pathways to care to reduce treatment delays in that ethnic group.

Thus far, whether first source of contact for help and referral source can independently determine DUP is not known. In addition, a previous qualitative study in Northern Malawi found that the socio‐cultural explanation of witchcraft and spirit possession was dominant in this area and influenced help‐seeking behaviours, with the majority of the participants reporting consulting traditional healers (THs) first, for diagnosis and to know who was responsible for the illness (Chilale, Silungwe, Gondwe, & Masulani‐Mwale, 2017). This help‐seeking tendency has been shown to prolong DUP in other populations (Al Fayez, Lappin, Murray, & Boydell, 2017; Tomita et al., 2015). However, that study in Northern Malawi used data from people's perceptions on psychosis to draw the conclusions, which did not take into account data from all possible contact places for help in the study area. It could be interesting to investigate from the patients themselves where exactly they sought for help before referral. That information could therefore substantiate the findings of that study to see which pathways to care contributed in the delays to effective treatment.

Therefore, the aim of this study was to explore referral sources to effective treatment in pathways to care among subjects with FEP from the same tribe in a community setting in Northern Malawi and examine the association between referral source and DUP and explore determinants of referral source; when adjusting for pathways to care, positive and negative symptoms, diagnosis and socio‐demographic characteristics.

## 2 METHODS

### 2.1 Setting

This study was conducted in the Northern region of Malawi with about 2 289 780 people (Government of Malawi, 2019) for which the Saint John of God (SJOG) community services provides community‐based mental health services. Although this population is multicultural, it is dominated by the Tumbuka ethnic group, which accounts for 57.3% of the population (Government of Malawi, 2019); hence the common language that is well understood by many people in this region is Tumbuka.

Noteworthy, in Malawi, the public health and mental health care system provides free health services at three levels: primary health care, secondary health care and tertiary health care (Ministry of Health (MoH) [Malawi] and ICF International, 2014). The primary health care comprises community initiatives, health posts, dispensaries, health centres and community/rural hospitals. Service delivery in health posts is mostly done by community‐based cadres such as health surveillance assistants (HSAs), community nurses, community‐based distributing agents, village health committees and other volunteers. Health posts refer cases to dispensaries or health centres and community/rural hospitals, where services are provided by medical assistants (with certificate in clinical medicine), HSAs, nurses and patient attendants. These cadres provide a range of mostly promotive and preventive services and some curative services. Secondary health care services are delivered by district hospitals, which are referral facilities for the primary health care and provide both inpatient and outpatient services for their target populations. The cadres at this level include medical assistants, clinical officers (with diploma in clinical medicine), Medical Doctors (with bachelor's degree in medicine and surgery—MBBS), nurses and patient attendants. Finally, tertiary health care services are provided by the central hospitals. These institutions also act as referral facilities for the district hospitals while providing services in their administrative regions. The cadres providing health services at this level include clinical officers, medical doctors, nurses, patient attendants and specialists such as paediatrician, gynaecologists, surgeons and dentists. Central hospitals also have the mandate to offer professional training, conduct research and provide support to the districts.

Therefore, this study area has at least six health centres, five district hospitals and one referral hospital, which also give mental health services, but insignificant due to inadequate resources. Thus, the community‐based mental health services provided by the SJOG community services are complementary. A high prevalence of mental disorders with long DUP among the Tumbuka ethnic group in the region motivated this study (Chilale, Banda, Muyawa, & Atipatsa, 2014). Ethical review was approved by the National Health Sciences Research Committee (NHSRC) of the Ministry of Health in Malawi (NHSRC/577).

#### 2.1.1 Study design

The sample of this study was collected, using specified inclusion criteria, from a group of patients who presented with psychosis to the Community Mental Health Care Team (CMHCT) of SJOG community services, during a pilot study of EIS for psychosis in Northern Malawi between June 2009 and September 2012.

First, the CMHCT visited the communities and health units to sensitize people on early signs and symptoms of FEP and an advertisement was made to receive and assess individuals for psychiatric disorders. The sensitization campaign mostly targeted health workers, community leaders, community based volunteers (CBVs), THs, families and religious leaders. In response to the sensitization campaign and advert, community members brought patients to the CMHCT for assessment.

Second, the CMHCT received or attended to people who were referred to them by the members of the community. In order to reach out to as many people as possible, a hotline was introduced on which members of the community could phone the CMHCT to attend to individuals with signs and symptoms of psychosis. The CMHCT sought collateral information from patients' relatives during assessment to confirm symptoms and diagnosis. An assessment was complete within 24 to 48 hours and then appropriate treatment was initiated. Depending on diagnosis, the following antipsychotic medications, commonly used in Malawi, were prescribed: oral drugs such as haloperidol tablets, chlorpromazine tablets and risperidone tablets; and injectable medications which stay in the body working for 4 weeks such as haloperidol decanoate, fluphenazine and depixol.

In this context, a patient was considered as receiving effective treatment when the patient tolerated the dose without or with very minimal side‐effects and, additionally, when the dose showed some positive effects on the targeted symptoms. On average, patients reached effective treatment within 3 to 4 weeks.

### 2.2 Subjects

Accordingly, the subjects of this study comprised some patients from the intervention described in the preceding section. Inclusion criteria were belonging to the Tumbuka ethnic group, consented to participate in the study; diagnosed of psychosis based on the Structured Clinical Interview for the Diagnostic and Statistical Manual of Mental Disorders‐Fourth Edition Text Revision (DSM‐IV‐TR) Axis I Disorders (SCID‐I) criteria (First, Spitzer, Miriam, & Williams, 2002); classified as having FEP by the CMHCT clinicians; and hardly had any antipsychotic medication before the intervention. Subjects with organic brain syndrome, drug abuse disorder, or learning disability were excluded from this study.

### 2.3 Study variables

Data were collected by research clinicians, who were well trained to use all the data collection tools for this study.

Psychiatric disorders were measured based on the DSM‐IV‐TR Axis I Disorders (SCID‐I) criteria (First et al., 2002). This tool is a semi‐structured diagnostic interview. It was adapted for use in this study because it has been widely used in psychiatric research studies, including cross‐national epidemiological and treatment studies (Gorman et al., 2004). In addition, this tool was used as gold standard when validating the Patient Health Questionnaire among 323 patients with type‐2 diabetes mellitus who attended two non‐communicable diseases (NCD) clinics in one of the 28 districts of Malawi (Udedi, Muula, Stewart, & Pence, 2019) and it was used when determining the prevalence of common mental disorders among South Africans seeking HIV testing (Kagee, Saal, Villiers, Laing, Sefatsa, & Bantjes, 2017) as well as when examining the content of delusions in a sample of South African Xhosa people with schizophrenia (Campbell et al., 2017). The introductory part was translated into Tumbuka language when taking the interviewers into the specific modules of the SCID‐I.

The main outcome variables for this study were pathways to care (initiation of help seeking, sources of contact, number of contacts and referral source to effective treatment) and DUP, which were identified retrospectively through interviews with patients and their relatives or significant others and using medical records wherever applicable. A standard instrument for measuring pathways to care has not been developed hitherto. Some previous studies found that pathways to care measures in first‐episode psychosis have been patients' narrative journeys through the healthcare system, which were diverse and varied across populations and this might have frustrated efforts to develop empirical standard instruments with optimal outcomes for measuring pathways to care (Singh et al., 2015; Singh & Grange, 2006). Therefore, this study adopted a pathways to care questionnaire from the Detection Education and Local Team project on early intervention in psychosis, in the Republic of Ireland, now known as the Dublin East Treatment and Early Care Team (DETECT) project (O'Callaghan et al., 2010; Renwick et al., 2008). This questionnaire was designed to elicit information on initiation of help seeking, reasons for help seeking, sources of contact for help, frequency of contact for each source of contact and referral source to effective treatment. DUP, defined as the length of time between onset of symptoms and receiving effective treatment, was measured using the Beiser scale (Beiser, Erickson, Fleming, & Iacono, 1993; Register‐Brown & Hong, 2014). Short DUP was defined as less than or equal to 6 months (≤6 months), while long DUP was defined as greater than 6 months (>6 months) (Addington et al., 2015; Kaminga et al., 2019; Ran et al., 2018).

Furthermore, positive and negative symptoms, which may cause people to seek for help, were assessed using the Scale for the Assessment of Positive Symptoms (SAPS) (Andreasen, 1984) and the Scale for the Assessment of Negative Symptoms (SANS) (Andreasen, 1983), respectively. These instruments were administered in Tumbuka language and then translated back into English to ensure that the same questions asked in different languages conveyed the same meaning. The SAPS comprises four domains, namely hallucinations, delusions, bizarre behaviour and positive formal thought disorder; whereas the SANS comprises five domains, namely affective flattening or blunting, alogia, avolition apathy, anhedonia asociality and attention. Each domain has a group of symptoms which characterizes it and each of these symptoms is rated from 0 (absent) to 5 (severe). The ratings of the symptoms under each domain are used to calculate the global rating of the severity of the domain on a scale from 0 (absent) to 5 (severe). The sum of the global scores of the domains was recorded for both SAPS and SANS.

Furthermore, socio‐demographic characteristics such as age at assessment, age at onset of symptoms, sex, marital status, level of education, employment and family history of psychiatric disorders were recorded.

### 2.4 Statistical analyses

Continuous variables were summarized using mean and SD when related data were normally distributed, otherwise quartiles were provided. Normality of continuous data was tested by the Shapiro‐Wilk's test (Shapiro & Wilk, 1965). Data related to the categorical variables were summarized using counts and percentages and the homogeneity of proportions across the categories was tested by the chi‐square (*χ* ^2^) test (Mendenhall, Beaver, & Beaver, 2009). Logistic regression analyses (Kirkwood & Sterne, 2003) were performed to examine the association between referral source and DUP and explore determinants of referral source; when adjusting for pathways to care, positive and negative symptoms, diagnosis and socio‐demographic characteristics. Important explanatory variables were tested for multicollinearity before performing multivariable logistic regression analyses (Sarkar & Rana, 2010). All the foregoing analyses were done in the Statistical Package for the Social Sciences Version 23.0 (IBM Corp., Armonk, NY) for Windows and all the statistical tests were two tailed with the significance level set at the 5%.

## 3 RESULTS

During the pilot EIS for psychosis in the study area, 400 people were referred to the CMHCT for assessment. Two hundred and one of the 400 people had psychosis and, among them, 140 subjects met the inclusion criteria. Noteworthy, descriptive statistics of continuous data that were not normally distributed were presented as lower quartile (Q1), median, upper quartile (Q3), minimum and maximum measurements. Age at assessment ranged between 18 and 65 years, with median, 33 years. Most of the subjects were male, had schizophrenia, were unemployed, had level of education more than primary, had long DUP and were married. Main sources of contact were general practitioner (GP), TH, religious advice (RA) and CBV. Majority did not initiate help seeking, had contact with GP or TH and had first contact with GP or TH. Distribution of subjects across the categories of referral source was not statistically different. One quarter of the subjects contacted GP or TH more than once. Overall, half of the subjects made at least two contacts for help prior to referral. Table 1 shows the details.

**Table 1:** Summary of sample variables measured

Study variable	*N* = 140
Age at assessment, in years	
Median (minimum‐maximum), Q1, Q3	33.0 (18.0‐65.0), 25.0, 42.8
Age at onset, in years	
Median (minimum‐maximum), Q1, Q3	28.0 (16.0‐65.0), 22.0, 36.0
DUP score in months	
Median (minimum‐maximum), Q1, Q3	12.5 (0.0‐370.0), 2.0, 75.5
SAPS total global score, mean (SD)	7.9 (3.4)
SANS total global score	
Median (minimum‐maximum), Q1, Q3	2.0 (0.0‐24.0), 0.0, 7.0
Initiation of help seeking, *n* (%)	
Self	12 (8.6)^a^
Other	128 (91.4)
Contact with GP, *n* (%)	
Yes	90 (64.3)^a^
No	50 (35.7)
Total number of times GP was contacted	
Median (minimum‐maximum), Q1, Q3	1.0 (0.0‐3.0), 0.0, 1.0
Contact with traditional healer, *n* (%)	
Yes	91 (65.0)^a^
No	49 (35.0)
Total number of times traditional healer was contacted	
Median (minimum‐maximum), Q1, Q3	1.0 (0.0‐4.0), 1.0, 1.0
Contact with religious advice, *n* (%)	
Yes	12 (8.6)^a^
No	128 (91.4)
Total number of times religious advice was sought	
Median (minimum‐maximum), Q1, Q3	0.0 (0.0‐1.0), 0.0, 0.0
Contact with community based volunteer, *n* (%)	
Yes	37 (26.4)^a^
No	103 (73.6)
Total number of times community based volunteer was contacted	
Median (minimum‐maximum), Q1, Q3	0.0 (0.0–1.0), 0.0, 1.0
Total number of contacts prior to referral	
Median (minimum‐maximum), Q1, Q3	2.0 (1.0‐4.0), 1.0, 3.0
First contact, *n* (%)	
GP	64 (45.7)^a^
Traditional healer	64 (45.7)
Religious advice	12 (8.6)
Referral source, *n* (%)	
GP	58 (41.4)
Community based volunteer	43 (30.7)
Traditional healer	39 (27.9)
DUP, *n* (%)	
≤6	49 (35.0)^a^
>6	91 (65.0)
Sex, *n* (%)	
Male	84 (60.0)^a^
Female	56 (40.0)
Marital status, *n* (%)	
Married or living with someone	68 (48.6)^a^
Separated/divorced/widowed	33 (23.6)
Single	39 (27.9)
Level of education, *n* (%)	
Primary or less	51 (36.4)^a^
More than primary	89 (63.6)
Employment, *n* (%)	
Employed	24 (17.1)^a^
Unemployed	116 (82.9)
Family history of psychiatric disorders, *n* (%)	
Yes	69 (49.3)
No	71 (50.7)
Diagnosis, *n* (%)	
Bipolar I disorder	12 (8.6)^a^
Major depressive disorder	1 (0.7)
Schizophrenia	94 (67.1)
Schizophreniform disorder	20 (14.3)
Schizoaffective disorder	6 (4.3)
Delusional disorder	1 (0.7)
Brief psychotic disorder	5 (3.6)
Psychotic disorder NOS	1 (0.7)

Abbreviations: DUP, duration of untreated psychosis; GP, general practitioner; Q1, lower quartile; Q3, upper quartile; SANS, scale for the assessment of negative symptoms; SAPS, scale for the assessment of positive symptoms.

a Homogeneity of proportions *χ* ^2^‐test, *P* value<0.05.

### 3.1 Association between referral source and DUP and determinants of referral source

The association between referral source and DUP and determinants of referral source were examined while adjusting for pathways to care, positive and negative symptoms, diagnosis and socio‐demographic characteristics. This was done because help seeking starts after observing or experiencing psychosis symptoms (positive or negative) and where to go for help or referred for treatment may depend on some socio‐demographic characteristics and diagnosis. Initially, univariate logistic regression analyses involving all the measured variables were performed to help identify explanatory variables in question. In this regard, long DUP was modelled in comparison with short DUP, while CBV or TH referral source was modelled in comparison with GP referral source and help initiated by others was modelled in comparison with help initiated by self. Table 2 shows the results.

**Table 2:** Univariate logistic regression models for DUP, referral source and help initiated by others

Variable	Univariate logistic regression analysis					
	Long DUP		Community based volunteer or traditional healer referral source		Help initiated by others	
	OR (95% CI)	*P* value	OR (95% CI)	*P* value	OR (95 CI)	*P* value
Initiation of help seeking						
Self	Reference		Reference		NA	NA
Other	0.92 (0.26‐3.23)	.899	2.11 (0.64‐7.02)	.222	NA	NA
Contact with GP						
No	Reference		Reference		NA	NA
Yes	0.23 (0.10‐0.54)	.001^a^	0.12 (0.05‐0.31)	.001^a^	NA	NA
Total number of times GP was contacted	0.27 (0.14‐0.53)	<.001^a^	0.25 (0.13‐0.49)	<.001^a^	NA	NA
Contact with traditional healer						
No	Reference		Reference		NA	NA
Yes	2.53 (1.23‐5.23)	.012^a^	10.28 (4.56‐23.18)	<.001^a^	NA	NA
Total number of times traditional healer was contacted	1.51 (1.03‐2.21)	.036^a^	2.38 (1.51‐3.76)	<.001^a^	NA	NA
Contact with religious advice						
No	Reference		Reference		NA	NA
Yes	0.51 (0.15‐1.66)	.262	0.053 (0.01‐0.42)	.006^a^	NA	NA
Total number of times religious advice was sought	0.51 (0.15–1.66)	.262	0.053 (0.01–0.42)	.006^a^	NA	NA
Contact with community based volunteer						
No	Reference		Reference		NA	NA
Yes	9.15 (2.64‐31.69)	<.001^a^	2.1 × 10^9^ (>0.00)	.999	NA	NA
Total number of times a community based volunteer was contacted	9.15 (2.64–31.69)	<.001^a^	2.1 × 10^9^ (>0.00)	.999	NA	NA
Total number of contacts prior to referral	1.26 (0.88‐1.81)	.208	1.77 (1.21‐2.58)	.003^a^	NA	NA
First contact						
GP	Reference		Reference		NA	NA
Traditional healer	2.19 (1.03‐4.65)	.041^a^	1.40 (0.68‐2.88)	.360	NA	NA
Religious advice	0.73 (0.21‐2.51)	.617	0.06 (0.01‐0.51)	.010^a^	NA	NA
Referral source						
GP	Reference		NA	NA	NA	NA
Community based volunteer	13.81 (4.36‐43.80)	<.001^a^	NA	NA	NA	NA
Traditional healer	3.61 (1.51‐8.62)	.004^a^	NA	NA	NA	NA
Sex						
Female	Reference		Reference		Reference	
Male	1.05 (0.52‐2.14)	.885	0.86 (0.43‐1.72)	.674	2.26 (0.68‐7.51)	.184
Marital status						
Married or living with someone	Reference		Reference		Reference	
Separated/divorced/widowed	2.19 (0.86‐5.55)	.099	1.98 (0.80‐4.90)	.138	2.36 (0.48‐11.63)	.290
Single	1.40 (0.62‐3.19)	.423	0.71 (0.32‐1.56)	.389	5.80 (0.71‐47.61)	.102
Level of education						
More than primary	Reference		Reference		Reference	
Primary or less	2.31 (1.07‐5.01)	.033^a^	2.99 (1.41‐6.36)	.004^a^	1.16 (0.33‐4.06)	.816
Employment						
Employed	Reference		Reference		Reference	
Unemployed	2.63 (1.07‐6.42)	.034^a^	4.44 (1.70‐11.59)	.002^a^	2.70 (0.74‐9.82)	.132
Family history of psychiatric disorders						
No	Reference		Reference		Reference	
Yes	1.49 (0.74‐3.00)	.265	0.75 (0.38‐1.48)	.408	0.67 (0.20‐2.23)	.671
Diagnosis						
Other	Reference		Reference		Reference	
Schizophrenia	10.35 (4.57‐23.48)	<.001^a^	3.82 (1.82‐8.03)	<.001^a^	0.38 (0.08‐1.82)	.227
SANS total global score	1.20 (1.08‐1.33)	.001^a^	1.07 (1.00‐1.15)	.053	1.23 (0.98‐1.54)	.077
SAPS total global score	1.04 (0.94‐1.15)	.479	0.88 (0.79‐0.98)	.019^a^	1.25 (1.03‐1.52)	.023^a^
Age at assessment	1.03 (1.00‐1.07)	.049^a^	1.05 (1.01‐1.08)	.006^a^	0.99 (0.94‐1.04)	.685
Age at onset	0.97 (0.94‐1.01)	.119	1.00 (0.97‐1.03)	.915	0.98 (0.93‐1.03)	.448

Abbreviations: CI, confidence interval; DUP, duration of untreated psychosis; GP, general practitioner; NA, not applicable; OR, odds ratio; SANS, scale for the assessment of negative symptoms; SAPS, scale for the assessment of positive symptoms.

a *P* value < .05.

In order to select explanatory variables for the multivariable regression analyses, all respective variables in Table 2 which were statistically significant (*P* < .05) and those that yielded *P* values of less than .1 (Hosmer & Stanley, 2000) were considered and then examined for multicollinearity. Paired comparisons indicated that contact with GP correlated with total number of times GP was contacted (*r* = 0.88, *P* < .001); contact with TH correlated with total number of times TH was contacted (*r* = 0.71, *P* < .001); contact with RA correlated with total number of times RA was sought (*r* = 1.0, *P* < .001); and contact with CBV correlated with total number of times CBV was contacted (*r* = 0.9, *P* < .001). Therefore, total number of contacts for each source was selected because not only does it identify contact source but also gives a measure of time spent with the source and this would make sense in assessing the dependent variables. Nevertheless, regarding DUP, some studies found that first contact source was associated with DUP (Bhui, Ullrich, & Coid, 2014), which was of great interest in this study, hence it was selected. However, first contact source correlated with the total number of contacts for each source (−0.75 ≤ *r* ≤ −0.66, or 0.51 ≤ *r* ≤ 0.67, *P* < .001), therefore, total number of contacts for each source was dropped in favour of first contact source, for DUP. However, when exploring variables associated with referral source, first contact source was dropped in favour of total number of contacts for each source. This made a lot of sense because first contact was part of the total number of contacts for each source contacted. Besides, overall number of contacts prior to referral was excluded because it correlated with total number of times TH was contacted (*r* = 0.77, *P* < .001). Also, age at assessment correlated with marital status and the former was selected because it was more associated with DUP (*P* = .049) than marital status (*P* = .099). In the end, the selected explanatory variables for DUP had weak pairwise correlations (−0.31 ≤ *r* ≤ 0.264, *P* < .001), low variance inflation factor (VIF) (1.12 ≤ VIF≤1.38) and a small condition index (13.66) corresponding to the smallest eigenvalue (0.026). Similarly, the selected explanatory variables for referral source had weak pairwise correlations (−0.36 ≤ *r* ≤ 0.26, *P* < .001), low VIF (1.09 ≤ VIF≤1.39) and small condition index (12.48) corresponding to the smallest eigenvalue (0.03). All these indicated low multicollinearity. Table 3 shows the results of the multivariable logistic regression models for long DUP (compared with short DUP), CBV or TH referral source (compared with GP referral source) and help initiated by others (compared with help initiated by self).

**Table 3:** Adjusted associations between referral source and DUP and determinants of referral source

Variable	Multivariable logistic regression analysis					
	Long DUP		Community based volunteer or traditional healer referral source		Help initiated by others	
	aOR (95% CI)	*P* value	aOR (95 CI)	*P* value	aOR (95% CI)	*P* value
Age at assessment	0.99 (0.95‐1.04)	.771	1.04 (0.99‐1.09)	.104	NA	NA
SAPS total global score	NA	NA	0.71 (0.60‐0.84)	<.001^a^	1.22 (1.00‐1.49)	.053
Total number of times GP was contacted	NA	NA	0.35 (0.15‐0.81)	.014^a^	NA	NA
Total number of times traditional healer was contacted	NA	NA	1.58 (0.94‐2.67)	.084	NA	NA
Total number of times religious advice was sought	NA	NA	0.05 (0.01‐0.52)	.012^a^	NA	NA
SANS total global score	1.15 (1.02‐1.30)	.024^a^	NA	NA	1.20 (0.95‐1.51)	.131
Level of education						
More than primary	Reference		Reference		NA	NA
Primary or less	1.26 (0.44‐3.59)	.667	1.42 (0.52‐3.85)	.490	NA	NA
Employment						
Employed	Reference		Reference		NA	NA
Unemployed	1.17 (0.36‐3.78)	.796	6.60 (1.67‐26.00)	.007^a^	NA	NA
Diagnosis						
Other	Reference		Reference		NA	NA
Schizophrenia	7.11 (2.70‐18.71)	<.001^a^	4.29 (1.46‐12.57)	.008^a^	NA	NA
First contact						
GP	Reference		NA	NA	NA	NA
Traditional healer	2.63 (0.99‐6.98)	.052	NA	NA	NA	NA
Religious advice	1.67 (0.35‐7.90)	.518	NA	NA	NA	NA
Referral source						
GP	Reference		NA	NA	NA	NA
Community based volunteer or traditional healer	4.23 (1.57‐11.36)	.004^a^	NA	NA	NA	NA

Abbreviations: aOR, adjusted odds ratio; CI, confidence interval; DUP, duration of untreated psychosis; GP, general practitioner; NA, not applicable; SANS, scale for the assessment of negative symptoms; SAPS, scale for the assessment of positive symptoms.

a *P* value < .05.

Unlike the models for DUP and referral source, the model for initiation of help seeking was not significant. The model for long DUP classified correctly (81/91)% = 89.0% of subjects with long DUP and (29/49)% = 59.2% of those with short DUP, with an overall correct success rate of (110/140)% = 78.6%. In addition, the positive predictive value, (81/101)% = 80.2%, indicated that, given the associated attributes, the probability of being classified as long DUP is 80.2% for this model. Also, this model was an improvement on the intercept only model (likelihood ratio test: *χ* ^2^(8, *N* = 140 = 60.0, *P <* .001) and fitted well with observed group memberships (goodness‐of‐fit test: *χ* ^2^(8, *N* = 140= 9.2, *P* = .327). Likewise the model for CBV or TH referral source classified correctly (74/82)% = 90.2% of those referred by CBV or TH and (44/58)% = 75.9% of those referred by GP, with an overall correct success rate of (118/140)% = 84.3%. The positive predictive value of this model was (74/88)% = 84.1% implying that it has 84.1% success rate in predicting those referred by CBV or TH. This model was indeed an improvement on an intercept only model (likelihood ratio test: *χ* ^2^(8, *N* = 140)= 74.7, *P <* .001) and fitted well with observed group memberships (goodness‐of‐fit test: *χ* ^2^(8, *N* = 140) = 7.2, *P* = .512).

Results in Table 3 indicated that first source of contact did not independently determine DUP (*P* > .05). However, a unit increase in severity of negative symptoms increased DUP by 15%. In addition, having the diagnosis of schizophrenia was about seven times more likely to be associated with long DUP than other diagnoses and referral from CBV or TH was about four times more likely to be associated with long DUP than referral from GP. Furthermore, having the diagnosis of schizophrenia was about four times more likely to be associated with referral from CBV or TH than other diagnoses and being unemployed was about seven times more likely to be associated with referral from CBV or TH than being employed. On the contrary, a unit increase in the number of times RA was sought, GP was contacted and severity of positive symptoms reduced the likelihood of being referred by CBV or TH by 95%, 65% and 29%, respectively. Despite an increase in positive symptoms being associated with help seeking initiation by others in the univariable analyses; it was not associated with initiation of help seeking when adjusted for negative symptoms.

## 4 DISCUSSION

To the best of our knowledge this is the first study in Malawi to examine the association between referral source and DUP and explore determinants of referral source; when adjusting for pathways to care, positive and negative symptoms, diagnosis and socio‐demographic characteristics. Therefore, this study found that, although first source of contact did not independently determine DUP, referral from CBV or TH was independently associated with long DUP. Also, a unit increase in severity of negative symptoms increased DUP as well as having the diagnosis of schizophrenia, which was also associated with referral from CBV or TH. Additionally, being unemployed was associated with referral from CBV or TH. However, a unit increase in the number of times RA was sought, GP was contacted and severity of positive symptoms increased the likelihood of being referred by GP.

Like in a previous similar study (O'Callaghan et al., 2010), most subjects in this study did not initiate help seeking, which was associated with less severe positive symptoms, but this was not significant when adjusted for negative symptoms. Also, similar to some previous studies (Adeosun, Adegbohun, Adewumi, & Jeje, 2013; Sharifi et al., 2009; Tomita et al., 2015), most subjects in this study had first contact with GP or TH. Although first contact in pathways to care has been shown to be associated with DUP in a previous study (Bhui, Ullrich, & Coid, 2014), this study has shown that this variable could be a confounding factor with referral source. Therefore, more future studies investigating the association between first contact source and DUP should consider adjusting for factors, such as referral source to ascertain this result.

Considering the fact that socio‐cultural explanation of witchcraft and spirit possession was dominant in the study area (Chilale, Silungwe, Gondwe, & Masulani‐Mwale, 2017), a substantial proportion of subjects might have been kept in the home or with a TH for so long (Kauye, Udedi, & Mafuta, 2014) until the commencement of the pilot EIS for psychosis, during which the CBV influenced referrals by themselves or TH. This is the most likely reason why referrals from CBV or TH were associated with long DUP. Therefore, this result underscores the importance of awareness campaigns on psychosis and collaboration with TH in reducing DUP in this population (Max Birchwood et al., 2018; Kauye, Udedi, & Mafuta, 2014).

In addition, TH basically interpreted psychosis symptoms as related to bewitchment or spirit possession for which they provided medications according to their expertise. Also, it had been observed that only when they portrayed a violent behaviour were people with hallucinations considered as mentally ill to warrant medical attention, otherwise they were regarded as on the course to becoming a TH “Kuthwasa” (Chilale, Silungwe, Gondwe, & Masulani‐Mwale, 2017; Kaminga et al., 2019). In this regard, however, TH could not report failure. That is, they could still keep a patient with hallucinations in worse situations where the patient is extremely violent or restless. In which case, they would tie the patient to a tree and, sometimes, beat up the patient with the belief that this would enhance recovery. As regards hallucinations associated with Kuthwasa, they were believed as spirits communicating with a patient on various types of diseases and their medications. Thus, the TH would ask the patient or guardian to choose driving out the spirits from the patient, or let the patient continue communicating with the spirits. Depending on the choice of the patient or guardian, the TH would attempt to exorcize the patient, or let the patient continue communicating with the spirits to eventually become a TH. Therefore, these practices delayed patients to receiving effective treatment. Most negative symptoms do not lead to violent behaviour and because of that people could not see them as symptoms justifying medical attention, thus this may explain why an increase in the severity of negative symptoms prolonged DUP. However, a unit increase in the severity of positive symptoms reduced the chances of referral by CBV or TH perhaps because more severe positive symptoms may lead to unbearable violent behaviours, which even a TH cannot handle. Therefore, such individuals were quickly rushed to receive treatment from hospital and were more likely to be referred by a GP. Contrariwise, those with less severe positive symptoms would be thought of as were on the course of becoming a TH, so they ended up spending more time with TH for monitoring the situation and hence were more likely to be referred by CBV or TH. Also, acute or insidious onset of positive symptoms might have played a role in this phenomenon (Chien & Compton, 2008), but this is a subject for further investigation in this population. Consequently, awareness campaigns are necessary in the study area to help people recognize that some symptoms of psychosis are not violent in nature or may not have a direct impact on relatives or community but require to be intervened early to avoid continuous damage resulting from long DUP (Marshall et al., 2005; Penttilä, Jääskeläinen, Hirvonen, Isohanni, & Miettunen, 2018).

Generally, long DUP was found to be prevalent in this study population among the schizophrenia subjects and this was associated with lower level of education, poor insight, younger age at onset and at least one parent deceased in this group (Kaminga et al., 2019). Therefore, this group might have stayed for so long without seeking for help either because of the foregoing factors, or because they sought help from TH on the basis of regarding the illness as a transition into becoming a TH. This may be the reason schizophrenia was associated with referral by CBV or TH in this study population. Consistently, some previous study also found that cases of schizophrenia were associated with community referrals to an early psychosis intervention for effective treatment (Ehmann et al., 2014). Thus, the schizophrenia group in this population requires special interventions, characterized by the associated risk factors, to reduce delays for effective treatment.

Also, this study found that those who were unemployed were more likely to be referred by CBV or TH than those who were employed. Considering that access to the public mental health services is free in Malawi, this phenomenon is more likely attributed to what other researchers have suggested that socioeconomic deprivation may explain inequalities in access to mental health services (Reichert & Jacobs, 2017). In this regard, it was suggested that a large proportion of people with FEP have already fallen out of education and employment at the time they come into contact with mental health services (Rinaldi et al., 2010), hence they may lack information about where to get effective treatment for mental health problems. In addition, with the provisions of health insurance schemes in Malawi, it could also be speculated that unlike those employed, most of the unemployed lack health insurance, perhaps due to lower household income and this may make medical services not easily accessible to them, particularly private medical services. Thus, when they miss out on public health services, they may look for other lower cost alternatives for help, such as TH or RA (Compton et al., 2009; Devi Thakoor et al., 2016; Large, Farooq, Nielssen, & Slade, 2008). Therefore, it is imperative to make mental health services readily available to the unemployed in the study area in order to reduce delays in receiving effective psychiatric treatment.

Furthermore, a unit increase in the number of times RA was sought and GP was contacted increased chances of referral by GP, which was associated with short DUP (Skeate, Jackson, Birchwood, & Jones, 2002). Moreover, just as in a previous study (Singh et al., 2015), faith based source of contact (RA) was not associated with delay in receiving effective treatment. Therefore, interventions to encourage people to contact GP or RA are warranted in this population as these may lead to shorter DUP and chances of better prognosis of the illness (Flora et al., 2017; Gavin et al., 2008; Power et al., 2007).

Some limitations need to be considered when interpreting the results of this study. This study focused on a specific ethnic group whose drivers of pathways to care may be different from those in other populations; therefore, this may preclude generalizing the results of this study to different populations. Also, pathways to care and DUP were measured retrospectively. Thus, there was a possibility of exaggerating these measurements. In addition, time between any two consecutive contacts prior to referral was not assessed because majority of the subjects could not remember precise dates they made the contacts. It could be interesting to see the impact of that on referral delays in this sample. Finally, the difference between the proportion of the employed subjects and the proportion of the unemployed subjects was huge, which may result into exaggerated estimations of its effects on DUP and referral source.

## 5 CONCLUSIONS

Referrals from sources other than GP increased DUP in the study population, so as having schizophrenia and a unit increase in the severity of negative symptoms. In addition, being unemployed and having schizophrenia increased the chances of referrals by CBV or TH. However, a unit increase in the number of times RA was sought, GP was contacted and severity of positive symptoms was more likely to result in referrals by GP. Therefore, awareness campaigns sensitizing people about the positive and negative symptoms of psychosis and the importance of seeking help from GP, other than from other sources, are needed in this population and a TH could be an important collaborator in this intervention. Also, improvement of access to mental health services is necessary, particularly for the unemployed group in the study area. Future studies should consider adjusting for referral source when ascertaining first contact source as a predictor of DUP.

## Data Availability

The data that support the findings of this study are available on request from the corresponding author. The data are not publicly available due to privacy or ethical restrictions.
